# Perspectives of HIV specialists and cardiologists on the specialty referral process for people living with HIV: a qualitative descriptive study

**DOI:** 10.1186/s12913-022-08015-0

**Published:** 2022-05-09

**Authors:** Charles Muiruri, Amy Corneli, Linda Cooper, Carrie Dombeck, Shamea Gray, Chris T. Longenecker, Eric G. Meissner, Nwora Lance Okeke, April C. Pettit, Teresa Swezey, Joseph Vicini, Gerald S. Bloomfield

**Affiliations:** 1 Department of Population Health Sciences, Duke University School of Medicine, 215 Morris St., Suite 210, Durham, NC 27701 USA; 2 Duke Global Health Institute, Duke University, Durham, NC USA; 3 Duke Clinical Research Institute, Duke University School of Medicine, Durham, NC USA; 4 Division of Infectious Diseases, Medical University of South Carolina, Charleston, SC USA; 5 Department of Medicine, Duke University School of Medicine, Durham, NC USA; 6 Department of Medicine, Case Western Reserve University School of Medicine, Cleveland, OH USA; 7 Department of Medicine, Vanderbilt University Medical Center, Nashville, TN USA

**Keywords:** Cardiology, HIV, HIV specialists, Specialty referral, Qualitative research

## Abstract

**Background:**

Cardiology care may be beneficial for risk factor management in people living with HIV (PLWH), yet limited information is available about the referral process from the perspectives of HIV specialists and cardiologists.

**Methods:**

We conducted 28 qualitative interviews at academic medical centers in the United States from December 2019 to February 2020 using components of the Specialty Referral Process Framework: referral decision, entry into referral care, and care integration. We analyzed the data using applied thematic analysis.

**Results:**

Reasons for cardiology referral most commonly included secondary prevention, uncontrolled risk factors, cardiac symptoms, and medication management. Facilitators in the referral process included ease of referral, personal relationships between HIV specialists and cardiologists, and close proximity of the clinic to the patient’s home. Barriers included lack of transportation, transportation costs, insurance coverage gaps, stigma, and patient reluctance.

**Conclusions:**

Our results will inform future studies on implementation strategies aimed at improving the specialty referral process for PLWH.

**Trial Registration:**

ClinicalTrials.gov Identifier: NCT04025125.

**Supplementary Information:**

The online version contains supplementary material available at 10.1186/s12913-022-08015-0.

## Background

Widespread availability of antiretroviral therapy (ART) has reduced AIDS-related mortality and improved survival for people living with HIV (PLWH) [1–3]. These improvements in longevity have led to an increased risk for other chronic conditions, including cardiovascular disease (CVD), in PLWH. Nearly half of PLWH in the United States (U.S.) are 50 years of age or older, the age after which cardiac events become more frequent, and CVD now accounts for approximately 15% of deaths among PLWH [2, 4, 5]. Large cohort studies have identified nearly twice the risk of incident acute myocardial infarction in PLWH compared with the general population [6, 7]. Despite this higher CVD risk and incidence, PLWH are less likely than persons not living with HIV to receive optimal CVD screening, diagnosis, and treatment [8–13]. Therefore, understanding and optimizing CVD prevention and care is urgently needed for PLWH [14, 15].

Determinants of optimal CVD care include factors at the patient, provider, and healthcare system levels. For example, PLWH may have a low perceived risk for CVD and may confront barriers associated with cost in accessing specialty care [16–18]. Compared with demographically similar cohorts in traditional primary care clinics, PLWH treated in HIV specialty clinics are less likely to be prescribed medications appropriate for coronary artery disease risk reduction [19]. In addition, compared with patients co-managed by a primary care provider and infectious disease (ID) clinician, PLWH managed by ID clinicians only are less likely to meet hypertension clinical guideline goals [13]. Physician compensation tied to relative value units has also been found to disincentivize HIV specialists from CVD prevention efforts [20]. These factors have resulted in heterogeneity in how care providers interact with the specialty referral process.

The value of specialty cardiology care for patients with established CVD (secondary prevention) is widely accepted, but referral for primary prevention and risk factor management may also be of benefit [21]. Among women of low socioeconomic status, Gilstrap et al. reported that use of interdisciplinary care teams, including a cardiologist, resulted in reduced prevalence of metabolic syndrome from 65 to 28% within 2 years [22]. This improvement was largely driven by improved blood pressure and cholesterol control. Further, patients in the general population who are seen by cardiologists are 2 times more likely to be prescribed recommended medication doses for primary prevention of dyslipidemia compared with patients who are seen by primary care physicians [23]. Although models that promote shared responsibilities for CVD management between non-HIV care providers and HIV care providers exist [13, 24, 25], optimal practices to improve use of specialty referrals for PLWH has not been rigorously studied.

To optimize and improve use of specialty cardiology care for PLWH, a deeper understanding of the implementation of the specialty referral process, including barriers preventing integrated care between a referring provider and cardiologist, is needed. The objective of this study was to identify reasons for cardiology care referrals for PLWH and barriers and facilitators of the referral process from the perspectives of HIV specialists and cardiologists.

## Methods

### Study design and participants

We conducted a qualitative descriptive study using in-depth interviews (IDIs) as part of the Pathways to Cardiovascular Disease Prevention and Impact of Specialty Referral in Under-Represented Racial and Ethnic Minorities with HIV (PATHWAYS) study (NCT04025125) [26, 27]. Qualitative methods were used to illuminate experiences on the referral process to provide a better understanding of the observed outcomes in quantitative published research [21–23] from the actors involved to support future research [28]. Participants of the study were from Duke University Medical Center (DUMC), Vanderbilt University Medical Center (VUMC), Case Western Reserve University (CWRU), and Medical University of South Carolina (MUSC); one site had integrated CVD and HIV care. Participants were purposively selected, and HIV specialists were eligible to participate if they were ID physicians, general internists, or advanced practice providers who had an active panel of PLWH under their care [29]. Cardiology providers were eligible to participate if they were a physician or advanced practice provider who provided care to at least 1 PLWH in the past 3 years. To increase participation, site principal investigators at DUMC, VUMC, CWRU, and MUSC presented the study goals at their respective institutions’ HIV clinics and cardiology divisions and provided contact information for the study personnel for those willing to participate. A $50 gift card was provided to participants.

This study was approved by the DUMC Institutional Review Board (IRB) as IRB of record for VUMC, CWRU, and MUSC through a single institutional review board reliance agreement.

#### Data collection

Two trained interviewers who were not affiliated with the clinics conducted telephone IDIs with participants from December 12, 2019 to February 28, 2020. Interviewers administered a brief demographic survey followed by interview questions developed by the study team (Supplemental Files 1 and 2).

**Fig. 1:**
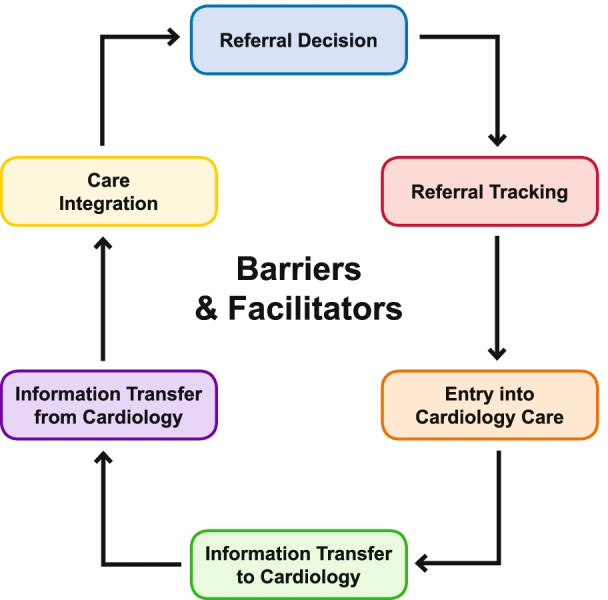
Cardiology referral framework

Our inquiry was informed by the Specialty Referral Process Framework [30], which we adapted for cardiology referrals. This framework incorporates paradigms of specialists’ clinical roles and continuity of care (Fig. 1). As described by Mehrotra et al., the referral process includes six components: referral decision, referral tracking, entry into specialty care, information transfer to specialists, information transfer from specialists, and care integration. During the IDIs with HIV specialists and cardiologists, we explored their referral decisions, entry into cardiology care, and experiences with care integration, particularly the barriers and facilitators they encountered.

#### Data analysis

We analyzed the IDI data using applied thematic analysis following a multi-stage deductive and inductive analysis approach [31]. First, all interviews were transcribed verbatim by a professional transcription service. Second, 2 analysts (CD and TS) independently applied structural codes (based on the specific interview topics) to the data using NVivo 12 (QSR International, Doncaster, Australia), a qualitative data analysis software program [32]. Third, analysts assessed inter-coder agreement in 14% of the transcripts (2 HIV specialists and 2 cardiologists) to ensure consistent application of the codes. Discrepancies in codes were resolved through discussion between coders. Edits were subsequently made to the structural codebook, and transcripts were re-coded as needed. Fourth, analysts reviewed the structural coding reports to identify and apply content codes (based on the overarching and emergent topics) to each structural coding report. Fifth, analysts wrote analytical summaries to describe the most frequently mentioned findings within the content codes as they related to reasons for referral, and referral barriers and facilitators. We used descriptive statistics to summarize the demographic data.

This work was conducted in line with the Consolidated Criteria for Reporting Qualitative Research (COREQ) checklist (Supplemental File 3) [33].

## Results

Twenty-eight providers participated in this study: 14 HIV specialists and 14 cardiologists. The majority (*n* = 16) of the providers were 40 to and 59 years of age. Slightly more than one-third (*n* = 10) of providers were female, and nearly all were White (*n* = 24) (Table 1). Nearly three-quarters (*n* = 11) of the HIV specialists cared for more than 100 PLWH each year. Most cardiologists cared for fewer than 20 PLWH per year. All cardiologists had completed a cardiology fellowship, whereas 10 HIV specialists had completed an ID fellowship (Table 2).

**Table 1:** Characteristics of study participants

Characteristics	*N* = 28
**Age**	
30–39	7
40–49	8
50–59	8
60–69	5
**Gender**	
Female	10
Male	18
**Race**	
White	24
Black or African American	1
Others	3
**Study sites**	
Duke University Medical Center	8
Case Western Reserve University	5
Medical University of South Carolina	7
Vanderbilt University Medical Center	8

**Table 2:** HIV provider and cardiologist characteristics

	**HIV Specialists demographics** ***N* = 14**	**Cardiologist demographics** ***N* = 14**
**Study sites**		
Case Western Reserve University	2	3
Duke University Medical Center	4	4
Medical University of South Carolina	4	3
Vanderbilt University Medical Center	4	4
**Training**		
Medical degree (MD)	12	14
Advanced practice providers	2	0
**Number of years providing care for PLWH**		
1–9	5	
10–19	4	
20–29	3	
> 30	2	
**Number of PLWH cared for each year**		
1–19	0	10
20–49	1	4
50–99	2	0
> 100	11	0
**Completed infectious disease (ID) fellowship (HIV providers) or cardiology fellowship (cardiologists)**	10	14

*PLWH* persons living with HIV

### Reasons for referral to cardiology care

The most common reasons PLWH were referred to cardiology care included the need for secondary prevention, uncontrolled CVD risk factors, suspected cardiac symptoms, and medication management.

#### Secondary CVD prevention

All HIV specialists stated they preferred to refer patients to a cardiologist for secondary or tertiary prevention when their patients had established CVD. Providers explained that they would refer patients who had cardiomyopathy, heart failure, arrhythmia, coronary artery disease, peripheral artery disease, valvular disease, and structural heart disease, among other conditions. Additionally, some HIV specialists expressed that cardiologists, instead of HIV specialists, should follow patients who have undergone cardiac procedures, such as bypass surgery or stent placement, and patients who have electrocardiogram (ECG) abnormalities or low ejection fraction on cardiac testing.

*…if they have a history of coronary artery disease or they’re having angina, then I don’t feel comfortable at all and that’s where I make sure that there’s a cardiologist that’s engaged. [HIV specialist, 16 years caring for PLWH]*

*…there's this thing in cardiology called an ejection fraction, which measures the strength of how well your heart’s beating basically. It's supposed to be around 65 percent. If I've got a patient who gets an echocardiogram and their ejection fraction is 25 percent, they get a referral – I'm not nuts about handling that patient. [HIV specialist, 10 years caring for PLWH]*

HIV specialists also described referring patients to cardiology care when they wanted assistance or advice with a patient who had been previously cared for by a cardiologist. The HIV specialists believed that they lacked CVD management experience or were not sufficiently updated on the current cardiology literature to manage PLWH with CVD on their own. The severity and complexity of the patient’s cardiac disease also played a role in the decision to refer. Providers expressed that if a patient with established disease began to show signs of increased severity, or if the case was complicated, they would be likely to refer.

Many cardiologists cited secondary prevention of established CVD as a primary reason for referral by HIV specialists. Heart failure, coronary artery disease, and previous percutaneous coronary interventions were the most common conditions noted by cardiologists. These referred patients were commonly followed long-term by the cardiologist.

#### Uncontrolled CVD risk factors

Almost all HIV specialists described referring their patients to cardiology care due to poorly or uncontrolled CVD-related conditions, such as hypertension or dyslipidemia. HIV specialists commented that they were generally comfortable making a first or second attempt at treating these conditions, as this fell within the bounds of their internal medicine training. However, if patients had tried several different medications without improvement or were on multiple simultaneous medications for hypertension or dyslipidemia and still not responding to treatment, they preferred to refer them to a specialist. HIV specialists frequently commented that they were not familiar with the newest therapies for these conditions, were not comfortable managing and titrating multiple CVD medications, or were unsure of how to proceed past second-line therapy. Some HIV specialists noted an informal “3-drug rule” for referrals, stating that if their patients needed more than 3 medications to control blood pressure, they would refer them to cardiology.

*Blood pressure, diabetes, lipid or dyslipidemia—I think those are the big things. But the caveat to that is I’ll usually only do it as an internal medicine doctor, level one is what I like to call it or freshman, maybe junior varsity. And when you have to go beyond that, I don’t have the long-term training to use …more alternative combinations with treatment for hypertension and dyslipidemia, for example. So, that’s when you take advantage of your consultants or other people doing stuff on cardiovascular disease, which is a big thing. …But when you start getting into tolerances and more complicated or more recent medications, I’m just not equipped. [HIV specialist, 23 years caring for PLWH]*

Most cardiologists described uncontrolled CVD comorbidities as an important reason why PLWH are referred by HIV specialists. Cardiologists noted that such patients did not necessarily need to be followed by them long-term, as they were often able to make recommendations for medication management, and patients could then be followed by their HIV specialists. However, for those with established CVD and uncontrolled CVD comorbidities, cardiologists tended to provide long-term follow-up and management of risk factors. A few cardiologists also shared that it was rare to receive referrals for first-line management of hypertension or lipids, noting that these therapies would typically have already been tried by the referring HIV specialist.

*We had a patient who had a murmur. They were referred to us. They had aortic stenosis that wasn’t severe enough to require surgery, but was still a short-term, needing some therapies in terms of blood pressure lowering, lipid lowering. They needed some surveillance imaging, so we set that program up in place and then that will transition from shorter-term care to longer-term care as we continue to monitor their valve disease over time. [Cardiologist, 24 years caring for PLWH]*

#### Suspected cardiac symptoms

Most HIV specialists stated that they referred their patients to cardiology for symptoms indicative of underlying cardiac disease, such as chest pain or unexplained edema. HIV specialists expressed varying degrees of comfort with performing diagnostic testing in the ID clinic, with some discussing that they would order an initial ECG and make a referral based on those results, whereas others noted that they would prefer for the initial workup to be conducted by a cardiologist.

*I would say any kind of chest pain we’d automatically refer. And you would make the distinction between something we think is emergent, which would probably be a trip to the ER, and something we think is sort of atypical chest pain, we would probably get an [ECG] here and read it, make sure there’s no acute process going on, and then we’d refer to cardiology. [HIV specialist, 27 years caring for PLWH]*

Numerous cardiologists noted that suspected cardiac symptoms were the main reason why PLWH were referred to them. Angina featured prominently in cardiologists’ narratives about referral. Cardiologists noted that they were frequently able to perform diagnostic testing to provide a diagnosis and/or a management plan, and the HIV specialist could provide long-term follow-up care.

*Sometimes it’s not risk factors that [patients] are referred for. Maybe it’s just a symptom that is concerning for the presence of cardiovascular disease. And then, I would see them, sometimes do some testing. Not invasive-wise to start with, but just stress test, or a heart monitor, or ultrasound, or something like that. And then, if that turns out favorably, then say, “All right, we investigated, everything else is in pretty good order cardiac wise, follow up as needed.” So, if someone’s having chest pain that the HIV specialist is concerned about, they might send them along for a further evaluation of that. And then, once that gets wrapped up, then it kind of becomes more of an as needed basis. [Cardiologist, 10 years caring for PLWH]*

#### Medication management

Both HIV specialists and cardiologists described issues related to medication management that could result in a PLWH being referred to cardiology. Some HIV specialists explained that a referral could be triggered once a patient began taking multiple medications for CVD. These providers expressed discomfort titrating CVD medications, citing less familiarity with the current guidelines for management, and feeling less comfortable managing highly specialized medications, such as certain blood thinners.

…in patients who are have multiple cardiac medications, titration of those – I would prefer that they be followed by a cardiologist. [HIV specialist, 4 years caring for PLWH]

Cardiologists stated that they would see these patients when HIV specialists had concerns about interactions between ART and CVD medications. All cardiologists cited statins in particular as having the potential to interact with ART. A few HIV specialists and cardiologists also shared that PLWH with a strong family history of CVD and an elevated risk of CVD would be candidates for referral to cardiology care.

### Facilitators of an optimal specialty referral process

HIV specialists identified a number of health system, structural, and interpersonal factors that facilitated the cardiology referral process for their patients. Health system factors focused on the ease of making referrals as supported by electronic health records.

*... the [electronic health records system] where you put in an ambulatory referral note. It’s very easy, and they’re pretty good. Some specialties you put in this referral and it seems like nothing ever gets scheduled. It’s just out there in no-man’s land for some reason, but cardiology seems like it does get scheduled and there seems like there’s enough cardiology clinics out there that my patient gets readily seen [HIV specialist, 16 years caring for PLWH]*

Other facilitators included having a referral assistant within the health system who could facilitate timely appointments and HIV specialists’ personal network of cardiologists or other specialists who were open to working with PLWH.

*Well, I think our system, having the referral specialists who can make sure that they’re covered and get them an appointment in a pretty timely manner, has been No. 1. [HIV specialist, 7 years caring for PLWH]*

*[W]e have a great working relationship with a lot of our cardiologists. We know them … in particular one comes to mind who is actually a geriatrician and cardiologist, and she sees a number of my patients who are aging. And I think she really does a great job with folks who have a ton of medication and maybe low health literacy, and she's very comfortable taking care of patients with HIV. I feel like we’ve got a good working relationship with a few of our cardiologists. [HIV specialist, 7 years caring for PLWH]*

HIV specialists also said they perceived that the proximity of the HIV and cardiology clinics to one another and/or to the patient’s home facilitated the referral process.

*Well, I think proximity for us [facilitates making cardiology referrals]. They [cardiologists] basically work right next door to our office here, and we’re all part of the same sub-specialty clinic that works within the hospital. [HIV specialist, 36 years caring for PLWH]*

### Barriers to an optimal specialty referral process

When asked about barriers to the referral process, providers focused their responses on patient-level barriers. Perceived obstacles included transportation, distance to travel, time, cost, and patient reluctance to see another provider. Some HIV specialists also described that perceived stigma and/or concerns about having to disclose their HIV status were barriers to specialty care referral for some patients.

#### Transportation and time barriers

Nearly all HIV specialists stated that problems with transportation and longer distances to the cardiology clinic were significant barriers to patients completing specialty referrals; many providers shared that this was the primary barrier. Time and financial costs were also described as barriers by many, and providers shared their perceptions that having to pay for public transportation, getting time off from work, taking time get to the clinic using public transportation, and/or trying to find a ride to a medical appointment made it difficult for patients to complete cardiology referrals. In addition, while transportation assistance was provided for HIV-related care, this assistance did not extend to cardiology care for PLWH.

*Transportation is a big [barrier] here. … public transportation can be an option for some of them, but often what we see is that they need to schedule a ride with the Disabled American Veterans van or they need to get a friend or a family member to bring them in, and so then you can imagine how that may play out if the friend or family member has a job for example. [HIV specialist, 36 years caring for PLWH]*

*Transportation, cost of transportation, especially if they are really low in terms of resources. And [name of city] has had this incredible economic boom where it’s just like one of the major boom cities in the country now. So people who used to be able to get Section 8 housing in town, that’s no longer available because all those places have been sold to make these ridiculously expensive condos. And so they’re living much farther out of town. And they depend on a really, really bad public transportation system. So without a doubt, number 1 barrier is getting there. [HIV specialist, 37 years caring for PLWH]*

#### Financial barriers

Many HIV specialists stated that high co-pays and deductibles, lack of insurance, limited support from financial support programs, and the cost of public transportation were financial barriers to referral care. A few providers explained that patients without adequate insurance may not keep their cardiology referral appointments. One provider elaborated that PLWH who received charity care and those with full insurance fared better in the referral process than those who were underinsured and had large out-of-pocket costs.

*I think cost is the main issue… it’s ironic that some of the best insured people have the most out of pocket costs, you know? So it’s hard to predict. Some of my patients only want to see me one time of year because of all the co-pays involved for the labs. [HIV specialist, 37 years caring for PLWH]*

#### Reluctance to see a new provider

A few HIV specialists stated that their patients were reluctant to see additional specialty providers. Providers explained that their patients had established close relationships with them over time and that some patients felt that it would not be easy to build rapport with a new provider.

*I think also it’s like the inconvenience of it, probably, another specialist. And then, you know, I think also I just for the most part get along really well with my patients and am pretty friendly with them and close with them. I talk to them a lot whether it’s through our EMR or whatever and they’re most likely not gonna get that with a specialist. They’re not gonna get that. So, they don’t wanna leave. [HIV specialist, 1 year caring for PLWH]*

#### Stigma and HIV status disclosure

Some HIV specialists perceived that patient concerns about stigma and status disclosures were barriers to specialty care referral, as patients were afraid about being judged at another clinic. HIV specialists noted that transgender patients, in particular, were concerned about negative reactions from staff members at the cardiology clinic (e.g., nurses, receptionists).

*I think it’s partly disclosing the HIV status. It’s another doctor, another provider to disclose that to. I think there are some concerns about that. Will they judge me? Will I know somebody there? It’s just that kind of thing. [HIV specialist, 1 year caring for PLWH]*

Table 3 provides other illustrative participant quotations.

**Table 3:** Other illustrative participant quotations by result categories

Categories	Illustrative Quotes
**1. Reasons for referral**	
Secondary CVD prevention	*…if they need stenting or a bypass then obviously, we need them to follow with a cardiologist. …If they have established CVD, I prefer that they're also under the care of a cardiologist. …Heart failure is another one, heart failure management is best done in conjunction with a cardiologist. [HIV provider, 1 year HIV experience]*
Uncontrolled CVD risk factors	*It’s more dependent on whether they’re responding to therapy or not. So, the discomfort comes in patients who are not responding to primary therapy. I think the best way to explain it would be that I am familiar with treatment of blood pressure, but in patients who don’t respond well to initial attempts to control blood pressure, I’m less comfortable with pursuing the next steps. …It’s more whether or not they’re responding to the therapies that I’m providing and whether I need help in that. Most likely, I need help in patients who …need blood pressure control and the blood pressure’s not getting under control. [HIV provider, 29 years HIV experience]*
Suspected cardiac symptoms	*I think that if they are having chest pain, I get nervous as all heck and so that is definitely a reason for me to refer someone to a cardiologist as well. If they’re having kind of chest pain where it’s atypical or not. [HIV provider, 16 years HIV experience]*
Medication management	*I am comfortable with most of them. Where it gets confusing is when they start needing more than one or two medicines. People who have really refractory hypertension and I start getting into four different blood pressure medicines and they’re still needing help. That’s where I start to get a little nervous that they should be going to a hypertension clinic for example. [HIV provider, 16 years HIV experience*]
**2. Facilitators of an optimal specialty referral process**	
	*Knowing that they have insurance is the biggest thing and the second biggest thing is having somebody to facilitate the referral for me…..* [*HIV provider, 10 years HIV experience]* *Well, if they live close-by, if they have insurance, all of those things make it much easier*. *[HIV provider, 13 years HIV experience]* *Well, I think for any referrals in the broadest sense, it helps if I know someone in the field who wants to do this type of care. So, some referrals are easy because I know the provider who likes to work with my patients and maybe has an interest in caring for people with HIV or just is generally eager to receive patients. Those are some of the things that make it easy. [HIV provider, 29 years HIV experience*]
**3. Barriers to an optimal specialty referral process**	
Transport and time barriers	*… [O]ne factor can be issues like – we don’t as a matter of rule for everybody provide let’s say transportation services. So, if someone has to come to clinic, sometimes it’s gonna cost them $1 or $2 or more to take a city transport. That actually pops into my brain as – we hear more about that in terms of socioeconomic limitations. It isn’t that – their visit would be covered. They would have no expense for their blood work or their visit, but they don’t have the $3 in order to get to the visit where my care would be free. [HIV provider, 23 years HIV experience]*
Financial barriers	*People do talk about insurance and whether or not it's covered. That's probably the most frequent response I get, is “Well, is it covered?” [HIV provider, 10 years HIV experience]*
Stigma, having to disclose HIV status	*I don't think I’ve had them [HIV patients] express serious concerns about seeing a cardiologist. I think sometimes they just – anytime there’s a new provider, more so I think in a primary care, they may not feel like they have the best relationship or it’s just another person they have to kind of get used to. I also will say that I think stigma is an issue for a number of patients. And they are concerned about going to a primary care provider locally when it’s gonna say HIV on their chart. So, that actually is a significant barrier for a number of people, particularly if they live in a small rural community. … [T]hey may not want to be seen really by anyone locally because they would feel compelled to disclose their HIV status as part of their non-HIV medical care. [HIV provider, 24 years HIV experience]*

*CVD* cardiovascular disease

## Discussion

This study addressed the reasons, barriers, and facilitators to engaging in specialty care for PLWH based on interviews with HIV and cardiology specialists from U.S. Midwest and Southern regions with a high prevalence of HIV and CVD. Cardiologists and HIV specialists at large academic medical centers in the U.S. identified several reasons for referring PLWH to cardiology care. Participants reported that PLWH were primarily referred for CVD medication management and if they had established CVD, suspected cardiac symptoms, or uncontrolled CVD-related conditions. Participants also identified barriers and facilitators of the referral process at the patient, provider, and health system level. Facilitators included ease of making referrals as supported by referral specialists and electronic health records, personal relationships between HIV specialists and cardiologists, and close proximity of the HIV and cardiology clinic to the patient’s home. As reported by the providers, perceived patient-level barriers that impeded the referral process included inefficient transportation to cardiology clinics, financial costs related to transportation, co-pays for specialty care, stigma, and patient reluctance to see additional providers. These findings contribute to the growing body of knowledge related to specialty care access for CVD by addressing these barriers and facilitators in PLWH, a population that has been understudied in this regard.

Since HIV specialists often act as de facto primary care providers for a majority of PLWH, they also act as “gatekeepers” with the responsibility of defining which PLWH require cardiology care [13, 20]. Established CVD requiring secondary CVD prevention and suspected cardiac symptoms were the primary reasons why HIV specialists referred PLWH to cardiologists. For patients with uncontrolled CVD-related conditions, HIV specialists reported that they first managed the conditions, including titration of CVD medications up to the provider’s level of comfort and confidence after which they would refer their patients to cardiologists mostly for medication management. In previous studies, HIV specialists were found to have low levels of comfort and confidence prescribing CVD-related medications, and the involvement of cardiologists in CVD medication management may be necessary [34, 35].

Future work is needed to determine the threshold of provider comfort prior to activating the referral system and the effectiveness of these referrals on CVD medication management and clinical inertia for PLWH.

Given that referrals were made based on an individual HIV specialist’s level of comfort and confidence in managing CVD-related conditions, heterogeneity likely exists in the type and frequency of referrals. Further, due to the lack of outcome data on the impact of referrals on CVD primary prevention and due to geographic disparities in HIV and CVD prevalence [36, 37], providers are likely to vary in their reasons for choosing to refer, with potentially negative impacts on clinical outcomes. Because referrals have considerable implications for PLWH, the healthcare system, and healthcare costs, future research is warranted to evaluate the effect of specialty referral and co-management practice patterns on CVD outcomes in PLWH and primary, secondary, and tertiary prevention based on regionally representative data. Further, alongside guidance on the integrated management of HIV and other non-communicable diseases, established guidelines are needed for the referral of higher-risk PLWH to cardiology specialists in the non-acute setting [38].

HIV specialists in this study reported that they rely on personal networks of cardiologists with experience caring for PLWH. As the population of PLWH continues to age and their risks for CVD increase, interventions are urgently needed to support cardiology care in this high-risk group [14]. Such interventions may include joint HIV/cardiology clinics, which have demonstrated efficacy in diagnosing and managing various cardiac conditions for PLWH [25]. This model may be particularly appropriate for PLWH given the barriers that were uncovered in this study related to transportation and stigma. Future research is necessary to adapt these interventions in different healthcare settings and evaluate their acceptability, fidelity, and appropriateness.

Barriers to the referral process were associated with the patient’s interactions with the health system outside the HIV clinic. Transportation challenges, including insufficient transportation infrastructure, have been found to be barriers to health services utilization for PLWH [39, 40]. Innovative approaches are needed to reduce the burden of travel times and costs of transportation for PLWH who require cardiology care, including ride-sharing, telemedicine, or e-consults [41, 42].

HIV specialists shared that PLWH may experience negative encounters at specialty care clinics due to stigma associated with their HIV diagnosis. A recent scoping review found that HIV stigma adversely affected PLWH in need of non-communicable disease care [43]. To reduce stigma, the authors recommended integrating non-communicable disease and HIV care. The few studies in this area are mixed on whether integration of HIV and non-communicable disease care leads to reduced stigma with data suggesting that the impact may differ at the healthcare facility and community levels [44]. Designing interventions that reduce stigma for PLWH who seek specialty care should be the focus of future research.

This study has several limitations. While internists were eligible to participate in this study, we were not able to enroll internists who may also refer patients to specialty care. Despite this limitation, our findings represent the experiences in the referral process for PLWH since the majority of these patients in the United States receive their care from providers with infectious disease specialty training [45]. We also did not interview other specialty care providers, such as nephrologists and endocrinologists, who HIV specialists refer their patients to for CVD risk factor management and who may have different views from those presented here. Given that there may be different organizational cultures within and between different specialties, future research should seek to evaluate if the findings in this study are different for other specialties. The study was also conducted at 4 large academic health systems and may not be generalizable to other healthcare systems. Barriers, referral patterns, and experiences may be different at other healthcare systems; therefore, future research should evaluate these differences for tailored interventions. Despite these limitations, participants of this study came from healthcare systems in the Midwest and Southern U.S. where the prevalence of HIV and CVD is high. The perspective of patients was beyond the scope of this inquiry, which focused on provider perspectives of specialty referral, but patient perspectives are critical and will be included in our future work.

## Conclusions

In conclusion, our findings can inform the development and evaluation of future interventions aimed at improving the specialty referral process for PLWH. This study brings the voices of HIV and cardiology providers to the forefront of the discussion on caring for other comorbidities in PLWH, which will aid in future intervention design to optimize CVD management in this high-risk population.

## Supplementary Information

**Additional file 1: Supplemental file 1.** Questions Guide for Interviews with HIV Providers Version 2.1.

**Additional file 2: Supplemental file 2.** Questions Guide for Interviews with Cardiologists Version 1.2.

**Additional file 3: Supplemental file 3.** Consolidated Criteria for Reporting Qualitative Research (COREQ) Checklist.

## Data Availability

Data are available at the corresponding author’s institution but are not publicly available due to participant confidentiality.

## Abbreviations

HIV: Human immunodeficiency virus
PLWH: Persons Living With HIV
CVD: Cardiovascular Disease
