# Supplementary material for: Perspectives of HIV specialists and cardiologists on the specialty referral process for people living with HIV: a qualitative descriptive study
Source: BMC Health Serv Res. 2022 May 9;22:623. doi: 10.1186/s12913-022-08015-0 (PMC9082896; doi:10.1186/s12913-022-08015-0)
Supplement: Supplementary file 1 — Additional file 1: Supplemental file 1. Questions Guide for Interviews with HIV Providers Version 2.1. [file 12913_2022_8015_MOESM1_ESM.docx]

**Questions Guide for Interviews with HIV Providers**

**Version 2.1**

| 1. Interviewer Name |  |
| --- | --- |
| 1. Participant ID# |  |
| 1. Interview Date (mm/dd/yyyy) | \|___\|___\|/\|___\|___\|/\|___\|___\|___\|___\| |
| 1. Participant agrees for interview to be digitally recorded | Yes 🞏  No 🞏 |
| 1. Time Interview Began (hh:mm) | \|___\|___ \| : \|___\|___\| am/pm |
| 1. Time Interview Ended (hh:mm) | \|___\|___\| : \|___\|___\| am/pm |

**Step 1:** Complete Q1-3 above before starting the interview.

**Step 2:** Introduce yourself at the beginning of the interview. Thank participant for taking part in the interview.

**Step 3:** Read “information about the study” below to participant.

**Step 4:** Ask for the participant’s permission to record interview. Tick appropriate box in Q4 above.

**Step 5:** Turn on audio recorder if permitted. Document time interview begins in Q5 above, and conduct interview.

**Step 6:** At the end of the interview, thank the participant and ask if she/he has any further questions. Document time interview ended in Q6 above.

**Step 7:** Collect reimbursement information and document appropriately.

**I. Information about the study**

Hello, I’d like to thank you for your time and interest in our research project. [Introduce self] The purpose of this research is to find out about your experience with primary and secondary prevention of cardiovascular disease among People Living with HIV (PLWH) and how patient care is coordinated within and between HIV providers and other specialists including cardiologists.

Your opinions as an HIV provider are extremely valuable as we try to better understand factors that influence the provision of primary preventive and secondary CVD care for people living with HIV in clinical practice. This information will assist in designing interventions that will improve the quality of CVD care in this high risk population. We are interested in your ideas, comments and suggestions. Please feel free to share your thoughts and talk candidly during the discussion.

Do you have any questions before we begin?

***[If yes, answer the participant’s questions.]***

Are you okay with our conversation being audio recorded?

***[If no]*** That’s okay, I’ll take detailed notes as we talk.

COMPLETE DEMOGRAPHIC FORM BEFORE STARTING MAIN PART OF THE INTERVIEW—DO NOT TURN ON AUDIO RECORDER UNTIL AFTER COMPLETING THE FORM

***Interviewer script: Thank you for that information. OK, let’s get started on the main part of the interview.***

***[If participant agreed to be recorded, begin audio recording now.]***

**Section 1: Attention to and Decision-Making Process for CVD prevention, HIV Providers’ role and comfort in CVD prevention for PLWH**

***Interviewer script*:** *I’d like to start by talking about when CVD prevention discussions might occur with your patients living with HIV.*

In thinking about the past year, how often did you talk about CVD prevention among your patients with HIV?

- 1. In what situations did you bring up CVD prevention with your patients living with HIV?
  2. In what situations did you not mention CVD prevention with your patients living with HIV even though they were at risk for CVD?

1. You likely need to discuss many topics with your patients during the short period of time when you see them. Where does CVD fall in terms of a priority area given all the other topics that must be discussed?
   1. What makes you feel that way?
2. What CVD risk factors, if any, are you comfortable managing?
   1. Why those?

1. Which CVD risk factors, if any, are you not comfortable with managing?
   1. Why those?

1. Overall, how comfortable or uncomfortable are you in managing patients with established CVD?
   1. [**If comfortable**] Why are you generally comfortable managing patients with established CVD?
   2. [**If uncomfortable**] For what reasons are you generally not comfortable managing patients with established CVD?
      1. What do you think could be done to increase your comfort level in managing this type of care?
2. Finally, whose role do you think it is to provide CVD prevention care to people living with HIV?
   1. What makes you feel that way?
      1. To what extent, if any, is CVD prevention care dependent on the severity of patients’ CVD?
      2. In what situations, if any, can the ID provider serve as the sole provider of CVD preventative care?
      3. In what situations should the cardiologist be the sole CVD preventive care?
      4. What are the situations when both types of providers should provide care?

**Section 2: Domain topic #2— Engagement in CVD preventive care and provider’s sense of responsibility on patient adherence**

***Interviewer script:*** *Now let’s talk about the recommendations you give for CVD prevention.*

1. Do you feel you do or do not have the appropriate training to provide recommendations for promoting cardiovascular health among your patients?
   1. What makes you feel that way?
   2. [**If no**] What type of training do you feel would be beneficial?
2. What are some of the common recommendations you typically give to your patients with CVD risk factors? (e.g. hypertension, tobacco use, depression, dyslipidemias)
3. What do you do, if anything, to formally or informally assess patients’ compliance to your recommendations on reducing CVD risk? For example, recommendations on medication adherence, exercise, or eating healthy.

**Section 3: Domain topic #3 —** Referral to Cardiology care

***Interviewer script:*** *Now let’s talk about the process of referring your HIV patients to specialty CVD care.*

1. What are some reasons you would refer a patient to a cardiologist for CVD care?

**Probes:**

- **severity of disease**
- **multiple comorbidities (if so, which ones)**
  1. When referring do you consider the
     1. Additional cost associated with seeing a specialist?
     2. Patients’ socioeconomic status?
     3. Insurance status

1. How, if at all, are your patients involved in making the decision to be referred to a cardiologist?
2. How did patients react to your recommendation that they see a cardiologist?
3. What concerns, if any, have they expressed about being referred to a cardiologist?
4. What, if anything, did they say might be good about being referred to a cardiologist?
5. What, if anything, makes it easy to refer patients to a cardiologist?
6. What barriers, if any, have you patients experienced in keeping the referral appointment?
   - 1. How do you think those barriers could be overcome?
7. What type of follow-up, if any, is done with HIV patients to assess whether they followed through with your cardiology referral?
   - 1. [**If follow-up**] Do you ever encounter any challenges or difficulties in following-up with patients about whether the kept their referral appointment with the cardiologist?

ii. [**If no follow-up**] What do you think could be done to follow up with your patients to see if they kept their appointment with a cardiologist?

1. What recommendations do you have for improving the efficiency and effectiveness of the CVD referral process for people living with HIV?
2. Once your patient has entered CVD care, what questions, if any, about your patients do you get from the cardiologist?
3. What types of information, if any do you typically send to a cardiologist who is in the same health system as your practice?
   - 1. How does that differ, if at all, with the information you would typically send to a cardiologist external to your health system?
   1. How do you communicate that information?
4. How effective is this system?
5. [**If ineffective]** What suggestions do you have for improving communication?
   1. What information is sent to you by the cardiologist?
6. How is this information communicated to you?
7. How effective is this system?
8. [I**f ineffective**] What suggestions do you have for improving communication?
9. What do you do with that information once received?
10. What information do you want to receive that you currently do not?
11. How does the involvement of an additional provider—meaning the cardiologist—affect the routine care that you provide to your HIV patients? For example, do you feel it enhances the care you provide, or does it complicate your care in any way? Please explain.

- 1. How, if at all, do you and the cardiologist agree on a management plan for the HIV patient?

1. In what areas do you disagree?
2. What is your role in providing CVD preventive care to the patient?
3. What is the cardiologist’s role?
4. What factors contribute to the decision about your role and the cardiologist’s role? (Probe about the frequency in which the patient visits the provider and the cardiologist)
   1. Overall, which do you prefer? You lead CVD preventive care, the cardiologist leads preventive CVD care, or you co-manage the patient’s CVD care with the cardiologist?
5. What makes you feel that way?

**Section 5: Closing**

That’s the end of the questions that I have for you today.

Is there any other information you’d like to share?

I want to sincerely thank you for your time and for the helpful information that you provided.

Thank you very much.

TURN OFF RECORDER

COMPLETE PARTICIPANT COMPENSATION PAPERWORK.
