# Supplementary material for: Perspectives of HIV specialists and cardiologists on the specialty referral process for people living with HIV: a qualitative descriptive study
Source: BMC Health Serv Res. 2022 May 9;22:623. doi: 10.1186/s12913-022-08015-0 (PMC9082896; doi:10.1186/s12913-022-08015-0)
Supplement: Supplementary file 2 — Additional file 2: Supplemental file 2. Questions Guide for Interviews with Cardiologists Version 1.2. [file 12913_2022_8015_MOESM2_ESM.docx]

**Questions Guide for Interviews with Cardiologists**

**Version 1.2**

| 1. Interviewer Name |  |
| --- | --- |
| 1. Participant ID# |  |
| 1. Interview Date (mm/dd/yyyy) | \|___\|___\|/\|___\|___\|/\|___\|___\|___\|___\| |
| 1. Participant agrees for interview to be digitally recorded | Yes 🞏  No 🞏 |
| 1. Time Interview Began (hh:mm) | \|___\|___ \| : \|___\|___\| am/pm |
| 1. Time Interview Ended (hh:mm) | \|___\|___\| : \|___\|___\| am/pm |

**Step 1:** Complete Q1-3 above before starting the interview.

**Step 2:** Introduce yourself at the beginning of the interview. Thank participant for taking part in the interview.

**Step 3:** Read “information about the study” below to participant.

**Step 4:** Ask for the participant’s permission to record interview. Tick appropriate box in Q4 above.

**Step 5:** Turn on audio recorder if permitted. Document time interview begins in Q5 above, and conduct interview.

**Step 6:** At the end of the interview, thank the participant and ask if she/he has any further questions. Document time interview ended in Q6 above.

**Step 7:** Collect reimbursement information and document appropriately.

**I. Information about the study**

(Optional if already introduced in step 2). Hello, I’d like to thank you for your time and interest in our research project. The purpose of this research is to find out about your experience with primary and secondary prevention of cardiovascular disease among people living with HIV and how patient care is coordinated within and between cardiologists and the patients’ primary care providers and/or ID specialists*.* I’ll refer to cardiovascular disease as CVD throughout the interview.

Your opinions as a cardiologist are extremely valuable as we try to better understand factors that influence the provision of primary preventive and secondary CVD care in clinical practice for people living with HIV. The information you provide will assist in designing interventions that will improve the quality of CVD care in this high risk population. We are interested in your ideas, comments and suggestions. Please feel free to share your thoughts and talk candidly during the discussion.

Do you have any questions before we begin?

***[If yes, answer the participant’s questions.]***

Are you okay with our conversation being audio recorded?

***[If no]*** That’s okay, I’ll take detailed notes as we talk.

COMPLETE DEMOGRAPHIC FORM BEFORE STARTING MAIN PART OF THE INTERVIEW—DO NOT TURN ON AUDIO RECORDER UNTIL AFTER COMPLETING THE FORM

***Interviewer script: Thank you for that information. OK, let’s get started on the main part of the interview.***

***[If participant agreed to be recorded, begin audio recording now.]***

**Section 1: Attention to and Decision-Making Process for CVD prevention, HIV Providers’ role and comfort with CVD prevention for PLWH**

***Interviewer script*:** *I’d like to start by talking about the burden of CVD among persons living with HIV and how these patients receive CVD care*

1. In your opinion, should CVD be a concern for people living with HIV, or not?
   1. What makes you feel that way?
2. In your opinion, how much of a problem are modifiable CVD risk factors for people living with HIV?
   1. What makes you feel that way?

When trying to reduce the risk of CVD for people living with HIV, where do you believe emphasis should be placed?

**[*If not mentioned, probe on hypertension, tobacco use, obesity, dyslipidemias]***

- 1. What makes you feel that way?

1. In your opinion, whose role should it be to provide CVD prevention care to people living with HIV—you as the cardiologist, or the patient’s primary care physician or infectious disease provider?

**Be sure to probe about primary and secondary CVD prevention (managing risk factors to prevent a CVD event vs. preventing another event in patients with established CVD)**

- 1. What makes you feel that way?
  2. How does the severity of the patients’ CVD play a role, if at all, in determining whose role it is to provide CVD care to people living with HIV?
  3. What are situations in which the cardiologist should be the sole provider of CVD preventive care?
  4. What are situations in which the ID provider should be the sole provider of CVD preventive care?
  5. What are situations in which both the ID specialist and the Cardiologist should provide CVD preventive care?
  6. What are the situations in which the patient’s primary care provider, rather than their ID provider, should be the sole provider of CVD preventative care?

**Section 2: Engagement and comfort in CVD preventive care and provider’s sense of responsibility on patient adherence**

***Interviewer script:*** *Now let’s talk about your engagement and comfort in primary and secondary CVD prevention for people living with HIV.*

1. What are common CVD risk factors for which people living with HIV get referred to you?

***[If not mentioned, probe on hypertension, tobacco use, obesity, dyslipidemias****]*

What CVD risk factors do you feel comfortable managing among people living with HIV?

1. [**If comfortable**] Why are you generally comfortable with these risk factors?

What CVD risk factors are you less comfortable managing among people living with HIV?

- 1. For what reasons are you generally not comfortable managing CVD care for these patients?
  2. What do you think could be done to increase your comfort level in managing this type of care?

1. What are some of the common recommendations you typically give for your patients who are living with HIV and who have modifiable CVD risk factors?

***[Probe for each risk factor mentioned earlier: treatment for hypertension and dyslipidemias , tobacco cessation, diet and exercise***

- 1. How do these recommendations vary for people living with HIV who have established CVD and those who do not?
  2. When making these recommendations, what factors do you take into account that you wouldn’t otherwise consider when caring for patients who do not have HIV? ***[Probe about patients with and without an established CVD diagnosis]***
  3. What considerations, if any, do you give to the insurance status of patients living with HIV? ***[Probe about patients with and without CVD diagnosis]***
  4. What considerations, if any, do you give to drug-to-drug interactions, given that these patients are also taking ARVs? ***[Probe about patients with and without CVD diagnosis]***

1. Do you feel you do or do not have the appropriate training to provide recommendations for promoting CVD health specifically among patients living with HIV?

a. What makes you feel that way?

b. [**If no**] What type of training do you feel would be beneficial?

**Section 3: Domain topic #3 —** Specialty referral process

***Interviewer script:*** *Now let’s talk about the kinds of referrals you receive for your patients living with HIV and care coordination with their referring providers.*

1. Thinking about the source/s of referrals for patients living with HIV, are most of those referrals made by the patient’s primary care provider, their infectious disease provider, or a mix of the two?
   1. Are there differences in the types of referrals that you receive from primary care providers vs. infectious disease providers?
      1. [**If yes**] Please explain
   2. In what ways, if any, does the insurance status of patients living with HIV play a role in whether they are accepted as a cardiology referral?
2. Please think about the types of referrals you receive for people living with HIV. What are the typical reasons why people living with HIV are referred to you?
   1. Are they usually a one-time consult, procedural, short term, or long term?
      1. What is a one-time consult typically like?
      2. What is a procedural visit only typically like?
   2. Are referrals usually for primary or secondary CVD prevention or both?
      1. What does short-term preventive care look like?
         1. Please give an example of a referral you had recently.
      2. What does longer term preventive care look like?
         1. Please give an example you have had recently.
   3. Of all these types of care, which do you find to yield better CVD outcomes for your patients living with HIV?
      1. What makes you feel that way?
3. When patients living with HIV are referred to you, what type of information do you receive from the referring provider?
   1. How useful or not useful is that information?
      1. What makes you feel that way?
   2. What other information would you want to receive from the referring provider before your encounter with these patients?
      1. How would you use this other information?
4. What type of information do you send back to the ID specialist or the PCP while the patient is being followed by you?
   1. Why is this information important?
   2. How do you communicate information with the ID specialist or the patient’s PCP while the patient is being followed by you?
      1. How effective or not effective is this process?
         1. What makes you feel that way?
      2. [**If not effective**] What suggestions do you have to make this process better?
5. How, if at all, do you and the referring provider (ID specialist or PCP) agree on a management plan for the patient?
   1. Do you prefer co-management, with shared care, where you and the referring provider have equal overall CVD care of the patient—or—do you prefer co-management, where you are responsible for the overall CVD care of the patient?
      1. Why?
   2. Of these two options, what is done more frequently among your patients with HIV?
      1. How does this typically work?
      2. What do you think could be done, if anything, to make this process better?

1. Now please think about how and why you currently receive referrals for people living with HIV. What recommendations do you have for improving the efficiency and effectiveness of the referral process in order to achieve quality of care for this high risk population?

Probe about:

- the care the cardiologist provides
- the communication and coordination that occurs during the specialty care for PLWH.
  1. Why would this be helpful?

**Section 5: Closing**

That’s the end of the questions that I have for you today.

Is there any other information you’d like to share?

I want to sincerely thank you for your time and for the helpful information that you provided.

Thank you very much.

TURN OFF RECORDER

**COMPLETE PARTICIPANT COMPENSATION PAPERWORK for DUHS providers only.**

**Update provider payment tracking forms for the three other sites, upload new versions to Box, and notify site PI/study coordinator when provider interviews are completed.**
