# Supplementary material for: Perspectives of HIV specialists and cardiologists on the specialty referral process for people living with HIV: a qualitative descriptive study
Source: BMC Health Serv Res. 2022 May 9;22:623. doi: 10.1186/s12913-022-08015-0 (PMC9082896; doi:10.1186/s12913-022-08015-0)
Supplement: Supplementary file 3 — Additional file 3: Supplemental file 3. Consolidated Criteria for Reporting Qualitative Research (COREQ) Checklist. [file 12913_2022_8015_MOESM3_ESM.doc]

COREQ (COnsolidated criteria for REporting Qualitative research) Checklist

**Implementation Analysis of Perspectives of HIV Specialists and Cardiologists on the Specialty Referral Process for**

**People Living with HIV: A Qualitative Descriptive Study**

| Domain | Item Number | Comment | Reported on Page Number or N/A |
| --- | --- | --- | --- |
| **Domain 1: Research team and flexibility** | | | |
| *Personal Characteristics* | | | |
| Interviewer/Facilitator | 1 | Authors CD and TS conducted the individual interviews | 4 |
| Credentials | 2 | Lead and co-authors have experience in conducting qualitative health research. | 5 |
| Occupation | 3 | Lead author was a health services researchers, Co-authors CTL, GSB were cardiologisits, EGM, NLO, ACP, were HIV specialists with extensive research experience caring for PLWH | Title page |
| Gender | 4 | Six researchers conducting this work were female | Title page |
| Experience and Training | 5 | Interviewers, analysts, lead author and one co-author had training in conducting qualitative health research. | N/A |
| *Relationship with Participants* | | | |
| Relationship established | 6 | Interviewers CD and TS did not have any affiliations or relationship with participants in this study to ensure that the collection of data was unbiased. | 4 |
| Participant knowledge of the interviewer | 7 | Each participant was introduced to the interviewers and the study team at the beginning of each interview. The participant was informed of how the data would be used and the purpose of the researcher and the study. | 3-4 |
| *Interviewer characteristics* | 8 | Interviewers were described as trained qualitative researchers | 4 |
| ***Domain 2: Study Design*** | | | |
| *Theoretical framework* | | | |
| Methodological orientation and Theory | 9 | The Specialty Referral Process Framework was described in the manuscript and was used to lead the development of the interview guide and codebook development. | 4 |
| Participant selection | | | |
| Sampling | 10 | Participants were selected using purposive sampling methodology. | 4 |
| Method of approach | 11 | HIV specialists and cardiologists at the study sites were approached to participate | 4 |
| Sample Size | 12 | 28 providers were interviewed in the study. | 7 |
| Non-participation | 13 | 2 participant were not interviewed due to scheduling conflicts. | N/A |
| Setting | | | |
| Setting of data collection | 14 | Individual interviews were conducted over the phone | 4 |
| Presence of non participants | 15 | Interviewers and participants were the only people present at the time of the interview. | N/A |
| Description of sample | 16 | Sample characteristics are reported in the results section. | 5 |
| *Data Collection* | | | |
| Interview guide | 17 | The interview guide was developed using the Specialty Referral Process Framework. Interview guide was tested before data collection. | 4 |
| Repeat interviews | 18 | No repeat interviews were conducted. | N/A |
| Audio/visual recording | 19 | All interviews were audio recorded on a password protected device. Audio recordings were deleted after uploading to secure study folder. | N/A |
| Field notes | 20 | The interviewer wrote field notes and reflection during and after interviews. | N/A |
| Duration | 21 | Each interview varied in duration but average length was 30-45 minutes | N/A |
| Data saturation | 22 | Data saturation was not relevant in content analysis. | N/A |
| Transcripts returned | 23 | No transcripts were returned to participants. | N/A |
| **Domain 3: Analysis and Findings** | | | |
| *Data Analysis* | | | |
| Number of data coders | 24 | Each transcript was coded by two coders (CD, TS). | 5 |
| Description of the coding tree | 25 | Codes and definitions were established a priori and derived from the Specialty Referral Process Framework. | 5 |
| Derivation of themes | 26 | Themes were established and defined through data analysis. | 5 |
| Software | 27 | NVivo version 12 was used for data analysis. | 5 |
| Participant checking | 28 | Participants did not provide feedback on the identified themes. | N/A |
| Reporting | | | |
| Quotations presented | 29 | Quotations were selected to represent each identified theme. Participants were kept anonymous and each quotation is attributed to “Respondent’s specialty and years caring for PLWH” | 6 -15 |
| Data and findings consistent | 30 | There is consistency between data presented and findings. | 6-15 |
| Clarity of major themes | 31 | Yes, major themes are clearly identified in relation to the specialty referral process | 6-15 |
| Clarity of minor themes | 32 | Yes, minor themes are clearly identified in relation the major themes | 6-15 |

Developed from: Tong A, Sainsbury P, Craig J. Consolidated criteria for reporting qualitative research (COREQ): a 32-item checklist for interviews and focus groups. International Journal for Quality in Health Care. 2007. Volume 19, Number 6: pp. 349 – 357
